# Inhibitor of DNA Binding 3 Limits Development of Murine Slam-Associated Adaptor Protein-Dependent “Innate” γδ T cells

**DOI:** 10.1371/journal.pone.0009303

**Published:** 2010-02-19

**Authors:** Mihalis Verykokakis, Markus D. Boos, Albert Bendelac, Erin J. Adams, Pablo Pereira, Barbara L. Kee

**Affiliations:** 1 Department of Pathology, University of Chicago, Chicago, Illinois, United States of America; 2 Committee on Immunology, University of Chicago, Chicago, Illinois, United States of America; 3 Howard Hughes Medical Institute, University of Chicago, Chicago, Illinois, United States of America; 4 Department of Biochemistry, University of Chicago, Chicago, Illinois, United States of America; 5 Unité du Développement des Lymphocytes, Institute Pasteur and Intitut National de la Santé et de la Recherche Medicale INSERM U668, Paris, France; New York University, United States of America

## Abstract

**Background:**

Id3 is a dominant antagonist of E protein transcription factor activity that is induced by signals emanating from the αβ and γδ T cell receptor (TCR). Mice lacking *Id3* were previously shown to have subtle defects in positive and negative selection of TCRαβ^+^ T lymphocytes. More recently, *Id3* ^−/−^ mice on a C57BL/6 background were shown to have a dramatic expansion of γδ T cells.

**Methodology/Principal Findings:**

Here we report that mice lacking Id3 have reduced thymocyte numbers but increased production of γδ T cells that express a Vγ1.1^+^Vδ6.3^+^ receptor with restricted junctional diversity. These Vγ1.1^+^Vδ6.3^+^ T cells have multiple characteristics associated with “innate” lymphocytes such as natural killer T (NKT) cells including an activated phenotype, expression of the transcription factor PLZF, and rapid production of IFNg and interleukin-4. Moreover, like other “innate” lymphocyte populations, development of *Id3* ^−/−^ Vγ1.1^+^Vδ6.3^+^ T cells requires the signaling adapter protein SAP.

**Conclusions:**

Our data provide novel insight into the requirements for development of Vγ1.1^+^Vδ6.3^+^ T cells and indicate a role for Id3 in repressing the response of “innate” γδ T cells to SAP-mediated expansion or survival.

## Introduction

T lymphocytes bearing αβ or γδ T cell receptors (TCR) develop in the thymus from a common progenitor cell pool. Most cells in the adult thymus express the co-receptor molecules CD4 and CD8 and represent an intermediate stage in αβ T cell development that has undergone productive TCRβ rearrangement and is in the process of TCRα rearrangement. After expression of a functional TCRα CD4^+^CD8^+^ (double positive, DP) cells undergo negative or positive selection and become single positive (SP) cells [1]. In contrast, the earliest T cell progenitors, and γδ T cells, are CD4^−^CD8^−^ (double negative, DN) and can be divided into four stages based on expression of CD117 (c-*kit*) and CD25; DN1, (c-*kit* ^+^CD25^−^), DN2 (c-*kit* ^+^CD25^+^), DN3 (c-*kit*-CD25^+^) and DN4 (c-*kit*-CD25−) [2]. DN1 and DN2 cells are the most immature T cell progenitors and are not yet fully committed to T cell differentiation [3], [4]. Rearrangement of TCR loci initiates at the DN2 stage but is most prevalent in DN3 cells [5], [6]. DN3 cells that rearrange and express TCRβ undergo β-chain selection and progress to the DN4 stage before becoming DP [7]. In contrast, cells that rearrange functional *Tcrg* and *Tcrd* genes diverge from the αβ pathway and become DN γδ T cells [2]. The stage at which the αβ and γδ T cell lineages diverge remains controversial [8], [9], [10], [11], [12].

During ontogeny, the variable gene segments of the *Tcrg* and *Tcrd* genes are rearranged in ordered waves. The first wave occurs around embryonic day 13 and includes rearrangement of Vγ3 and Vδ1 and is followed by rearrangement of Vγ4 [13], [14] (Nomenclature according to [15]). These receptors contain limited diversity at the junction of the V, diversity (D), and joining (J) segments [16], [17] in part because terminal deoxylnucleotidyl transferase (TdT), a polymerase that adds non-templated nucleotides, is absent from embryonic cells [18], [19]. Consequently, the first γδ T cells express invariant Vγ3/Vδ1 or Vγ4/Vδ1 TCRs and home specifically to the epidermis or the epithelium of the reproductive tract and the tongue, respectively [20]. In contrast to the embryo, the adult thymus rearranges Vγ1.1, Vγ2 and Vγ5 and generates receptors with extensive junctional diversity, creating a highly diverse γδ TCR repertoire [21]. Interestingly, a subset of γδ T cells with an invariant Vγ1.1^+^Vδ6.3^+^ TCR has been described that resides in the adult thymus, spleen, and liver [22]. These γδ T cells develop from late embryonic precursors and expand during neonatal life [23]. Vγ1.1^+^Vδ6.3^+^ T cells share multiple characteristics with natural killer (NK) T cells including expression of the activation markers CD44, and NK1.1, and low expression of the immature T lymphocyte marker CD24. Moreover, both NKT and Vγ1.1^+^Vδ6.3^+^ T cells secrete IFNγ and IL4 rapidly after stimulation *in vitro* [24]. These findings led to the hypothesis that NKT and Vγ1.1^+^Vδ6.3^+^ T cells represent innate branches of the αβ and γδ T cell lineages, respectively [25]. The presence of an invariant receptor on these two T cell subsets is consistent with the hypothesis that the functional characteristics of these “innate-like” cells are determined in part via selection by endogenous ligands.

T cell development is intimately linked to activity of the E protein transcription factors E2A and HEB [26], [27], [28]. E proteins are essential at multiple stages of αβ T cell development and function in lymphocyte survival, proliferation and differentiation. Importantly, induction of E protein antagonists such as Id2 and Id3 appears to be critical for β-selection and positive selection of αβ T cells [29], [30], [31]. Cross-linking of CD3ε (a component of the TCR signalling complex) on DN3 thymocytes induces Id3 through a MAP kinase-dependent pathway [32]. Mice lacking *Id3* show mild defects in positive selection similar to those observed in mice lacking the Tec kinase Itk, which activates the MAP kinases Erk1 and Erk2 [33], [34]. MAP kinase signalling is also important for proper γδ T cell development and Id3 is highly expressed in γδ T cells, although published data suggest that Id3 is not essential for γδ T cell development in mice expressing the KN6 (Vγ2^+^Vδ5^+^) transgene [10]. Surprisingly however, it was reported that *Id3* ^−/−^ mice have an increased number of γδ T cells and it was hypothesized that Id3 functions in DN3 cells to prevent *Tcrg* or *Tcrd* rearrangement in cells expressing a functional TCRβ [35].

Here we report that the elevated number of γδ T cells in *Id3* ^−/−^ mice is a consequence of an expanded population of Vγ1.1^+^Vδ6.3^+^ T cells. *Id3* ^−/−^ Vγ1.1^+^Vδ6.3^+^ T cells, like their wild-type (WT) counterparts, primarily develop from late embryonic or neonatal progenitors rather than adult DN3 cells. These γδ T cells have many of the characteristics of NKT cells previously noted, and we confirm that *Id3* ^−/−^ Vγ1.1^+^Vδ6.3^+^ T cells express the transcription factor promyelocytic leukemia zinc finger (PLZF) protein [36], a molecular determinant of the NKT cell fate [37], [38] and their development required the Signaling lymphocyte adaptor molecule (Slam)-associated Adaptor Protein (SAP) [39]. Importantly, deletion of SAP overcomes all apparent thymic alterations in *Id3* ^−/−^ mice including the increased number of γδ T cells and the reduced thymic cellularity, whereas deletion of *Tcrd* had no effect on thymic cellularity. These observations indicate that Id3 plays a role in preventing expansion or survival of this SAP-dependent lymphocyte. Taken together, our data demonstrate that Id3 functions to limit the development of SAP-dependent “innate-like” γδ T cells.

## Results

### Development of CD4^+^ and CD8^+^ γδ T Cells in *Id3^−/−^* Mice

**Figure 1:**
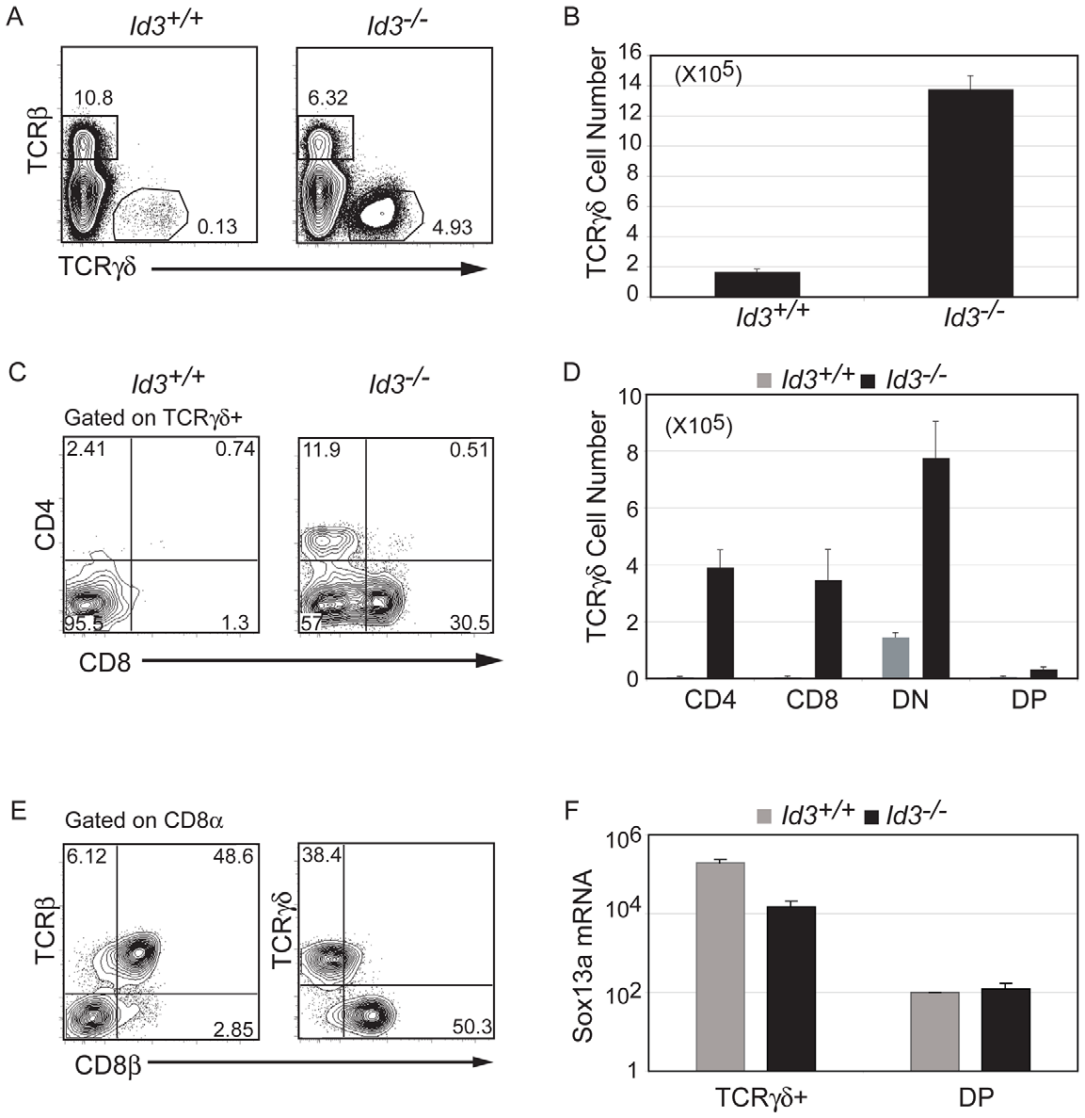
Altered γδ T cell development in *Id3^−/−^* mice. A) FACS analysis for TCRβ and TCRγδ on *Id3* ^+/+^ and *Id3* ^−/−^ thymocytes. The frequency of TCRβ^+^ cells and TCRγδ^+^ cells is shown. B) Number of TCRγδ^+^ cells in the thymus of *Id3^+/+^* and *Id3^−/−^* mice. Average +/− standard deviation was determined from >15 mice. p<0.0005. C) CD4 and CD8 expression on TCRγδ^+^ cells. D) Number of CD4, CD8, DN and DP TCRγδ^+^ cells in the thymus *Id3^+/+^* (grey) and *Id3^−/−^* (black) mice. Average +/− standard deviation was determined from >15 mice. p<0.0005 in all *Id3* ^+/+^ to *Id3* ^−/−^ comparisons. E) Analysis of *Id3^−/−^* CD8α^+^ thymocytes for TCRβ and CD8β (left panel) or TCRγ (right panel) and CD8β. The TCRβ^+^ cells express CD8β whereas TCRγδ^+^ cells are CD8β^−^ (right panel) and presumably CD8αα. F) QPCR for Sox13a mRNA in sorted *Id3^+/+^* (grey) and *Id3^−/−^* (black) TCRγ^+^ cells and DP thymocytes (standardized to *Hprt*). Bars are the average from 3 experiments +/− standard deviation.

While investigating the thymic phenotype of *Id3^−/−^* mice we discovered that the number of γδ T cells is increased by approximately 8–fold (range 3- to 15-fold) compared to *Id3* ^+/+^ mice (**Fig. 1A and B**). In contrast, the frequency of TCRβ^high^ cells was similar among *Id3* ^+/+^ and *Id3* ^−/−^ thymocytes, although the number of TCRβ^+^ cells is decreased in the absence of *Id3* since *Id3* ^−/−^ mice have a 3-fold decrease in thymocytes numbers (**Fig. 1A** and **Fig. S1**). Further analysis revealed that a large portion of *Id3^−/−^* TCRγ^+^ cells express CD4 or CD8 (**Fig. 1C**). Compared to *Id3^+/+^* mice, *Id3^−/−^* mice have an increased number of CD4 (80-fold) and CD8 (70-fold) TCRγ^+^ cells as well as DN (5.5-fold) and DP (5-fold) TCRγ^+^ cells (**Fig. 1D**). *Id3^−/−^* TCRγ^+^ cells express CD8 as a CD8αα homodimer as opposed to the CD8αβ heterodimer expressed by TCRβ^+^CD8^+^ cells (**Fig. 1E**). Importantly, *Id3^−/−^* TCRγ^+^ cells expressed significantly more mRNA for the transcription factor Sox13 than DP thymocytes indicating that these are bona fide γδ T cells [40] (**Fig. 1F**). In the spleen there is also a large population of TCRγ^+^ cells expressing CD4 or CD8αα that is markedly elevated compared to *Id3^+/+^* mice (**Fig. S2**). Taken together these data indicate that Id3 limits development of γδ T cells, in particular, γδ T cells expressing CD4 or CD8.

### *Id3^−/−^* γδ T Cells Have an Activated or “Innate-Like” Phenotype

**Figure 2:**
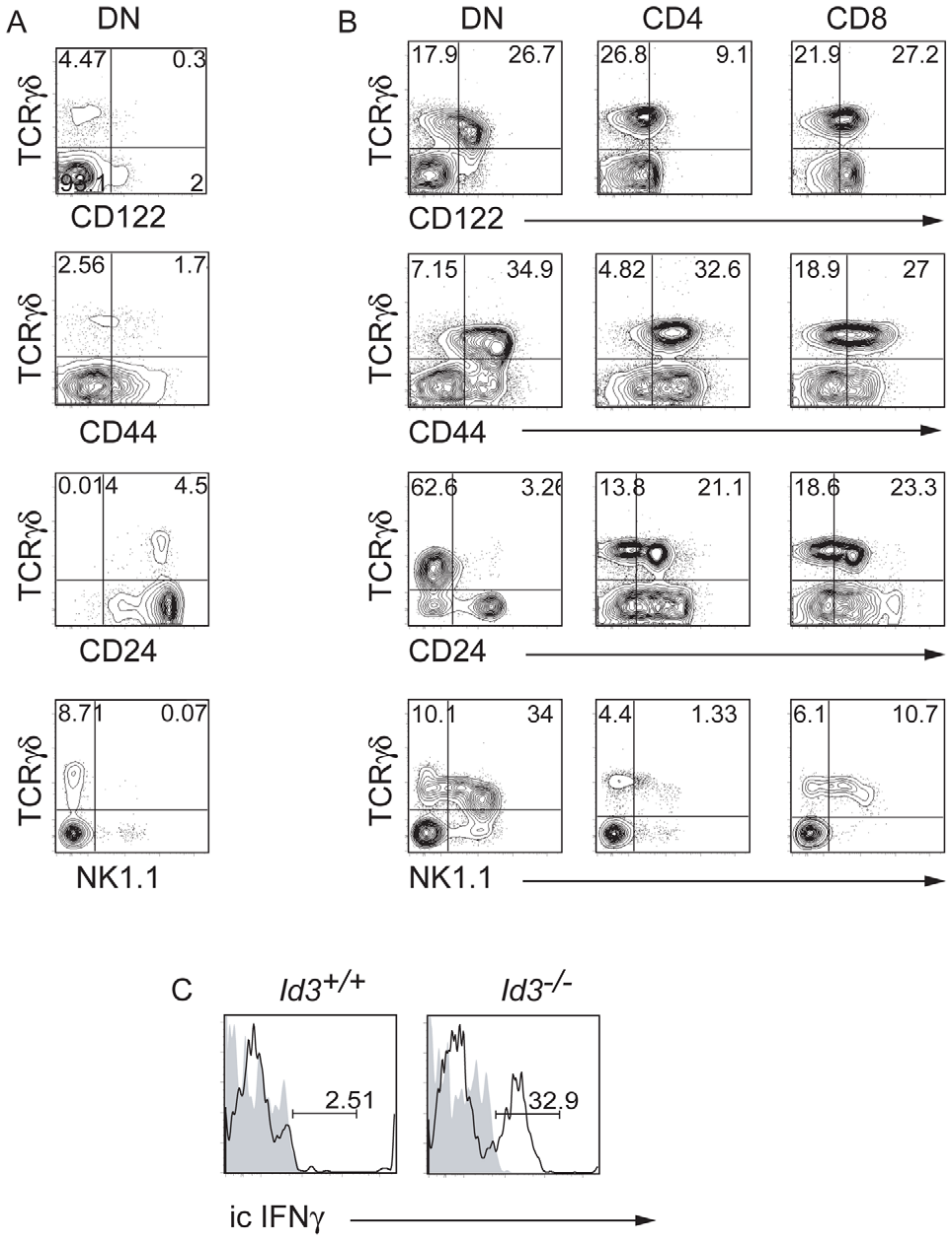
*Id3^−/−^* γδ T cells have an activated phenotype. FACS analysis of *Id3* ^+/+^ DN thymocytes (A) or *Id3* ^−/−^ DN, CD4 and CD8 thymocytes (B) for expression of TCRγδ and CD122, CD44, CD24 or NK1.1. The analysis shows that *Id3* ^−/−^ γδ T cells have markers of activation independent of expression of CD4 or CD8. Data are representative of >10 experiments. C) FACS analysis showing intracellular staining for IFNγ (open histogram) in *Id3^+/+^* and *Id3^−/−^* TCRγδ^+^ thymocytes 5 hours after stimulation with PMA and ionomycin. Shaded histogram shows isotype control. One of 3 experiments is shown.

In light of our observations that *Id3^−/−^* γδ T cells expressed CD4 and CD8, we characterized these cells for expression of multiple cell surface proteins. In the thymus, the majority of *Id3^−/−^* DN TCRγ^+^ cells had high expression of CD122, NK1.1 and CD44 and low expression of CD24 compared with *Id3^+/+^* TCRγ^+^ cells (**Fig. 2A and B**). This phenotype is associated with activation of αβ and γδ T cells [41], [42], [43]. Notably, a majority of the *Id3* ^−/−^ NK1.1^+^ TCRγ^+^ cells expressed TCRγ at low levels (**Fig. 2B**). A subset of γδ T cells expressing NK1.1 with low expression of TCRγδ is present in the spleen of WT mice and presumably represent activated γδ T cells [42]. Similar to *Id3* ^−/−^ DN TCRγδ^+^ cells, a portion of *Id3^−/−^* CD4 and CD8 γδ T cells had these activation markers, although the CD4 cells had lower levels of CD122 and only a small portion expressed NK1.1 (**Fig. 2B**). Therefore, our data indicate that a large portion of the γδ T cells in the thymus of *Id3^−/−^* mice have an activated phenotype. The DN, CD4 and CD8 γδ T cells in the spleen of *Id3^−/−^* mice also have an activated phenotype (**Fig. S3**).

A subset of CD122^+^ γδ T cells, which are thought to have encountered ligand in the thymus, produce IFNγ rapidly after in vitro stimulation [43]. To determine whether *Id3^−/−^* γδ T cells represent previously activated cells we tested their ability to make IFNγ after *in vitro* stimulation with PMA and ionomycin for 5 hours. Importantly >30% of *Id3^−/−^* TCRγ^+^ thymocytes produce IFNγ under these conditions. In contrast only 2.5% of *Id3^+/+^* TCRγ^+^ thymocytes produced IFNγ at this early time point (**Fig 2C**). Similarly, more than 50% of *Id3^−/−^* splenic TCRγ^+^ cells produced IFNγ (**Fig S3**). Interestingly, a subset of *Id3* ^−/−^ γδ T cells make both IFNγ and IL-4 (**Fig. S4**). Cytometric bead analysis revealed that *Id3* ^−/−^ γδ T cells also make more IFNγ, IL4, IL10 and IL13 than their WT counterparts after stimulation with anti-TCRγ (**Fig. S4**). Taken together, our data demonstrate that *Id3* ^−/−^ mice develop a large population of γδ T cells that show characteristics of previously activated cells.

### *Id3^−/−^* γδ T Cells with an Activated Phenotype Develop Early in Post-Natal Life

**Figure 3:**
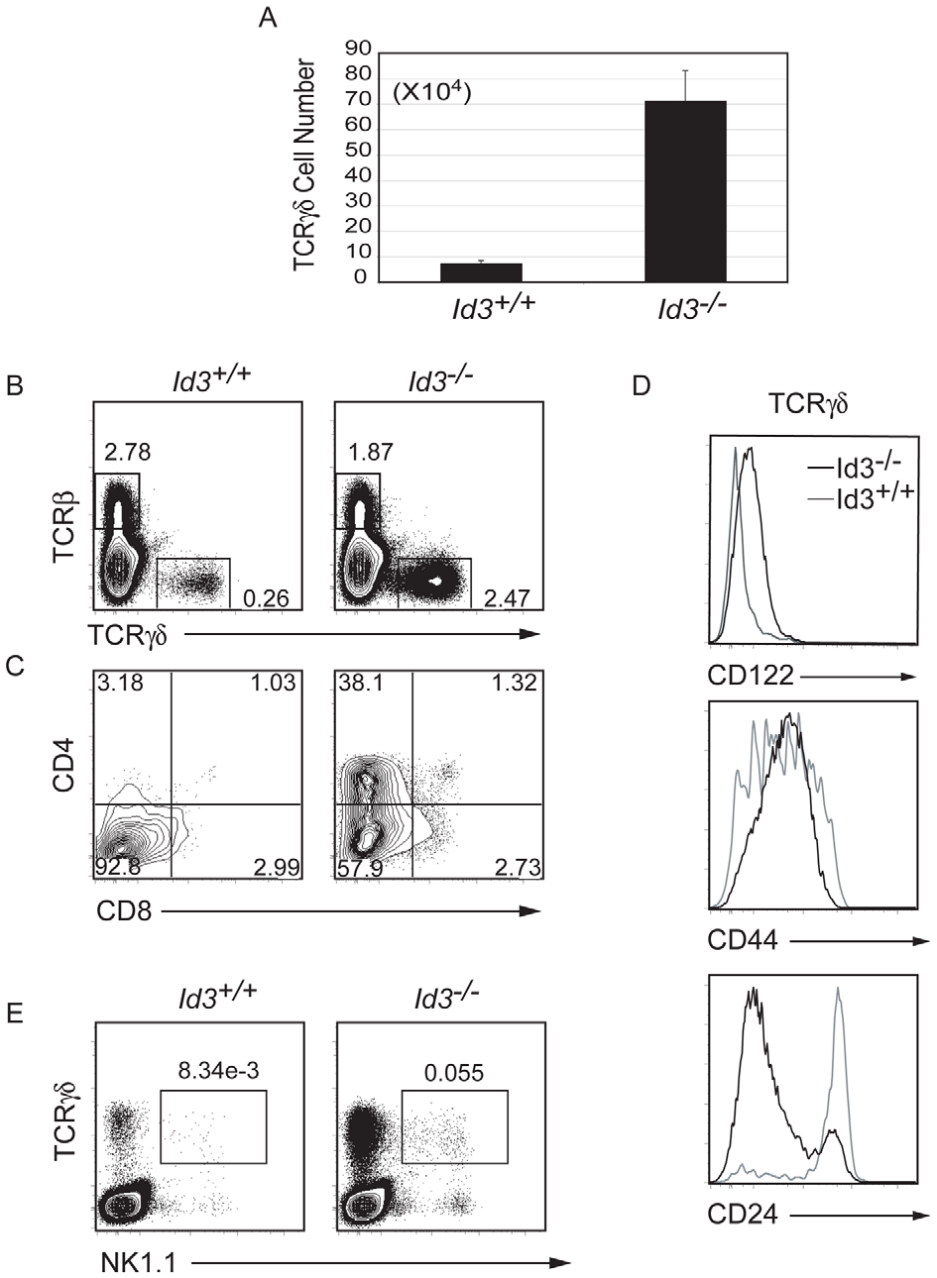
γδ T cells with an activated phenotype are present in *Id3^−/−^* neonates. A) γδ T cell numbers in *Id3^+/+^* and *Id3^−/−^* mice analyzed 7 days after birth. Average +/− standard deviation is derived from 6 mice. p<0.0005. B) TCRβ and TCRγδ expression on total thymocytes from 7 day old mice. C) Gated TCRγδ^+^ cells were analyzed for CD4 and CD8. D) Expression of CD122, CD44 or CD24 on TCRγδ^+^ thymocytes from 7 day old *Id3^+/+^* (grey) and *Id3^−/−^* (black line) littermates. E) TCRγδ and NK1.1 expression total thymocytes from the same pair of mice as in D.

To determine when during ontogeny *Id3* ^−/−^ γδ T cell numbers increase and when the activated phenotype becomes evident, we examined thymocytes from mice isolated 1 week after birth. At this stage of ontogeny, few thymocytes have left the thymus and therefore peripheral activation is unlikely to have impacted on thymocyte numbers or phenotype. Importantly, a 10-fold increase in TCRγ^+^ cells was observed in *Id3^−/−^* neonates and the aberrant expression of CD4 was already evident (**Fig. 3A, B and C**). Moreover, *Id3* ^−/−^ neonatal γδ T cells had an activated phenotype similar to that observed in the adult *Id3^−/−^* thymus (**Fig. 3D and E**), although only a small subset of these cells were positive for NK1.1. Taken together, our data indicate that Id3 limits the development of γδ T cells with an activated phenotype in neonatal mice. Our data also indicate that the activated phenotype of *Id3* ^−/−^ γδ T cells likely occurs within the thymus rather than as a consequence of peripheral activation since few thymocytes have left the thymus within the first week after birth [44].

### The Majority of *Id3^−/−^* γδ T Cells Express Vγ1.1 and Vδ6.3

**Figure 4:**
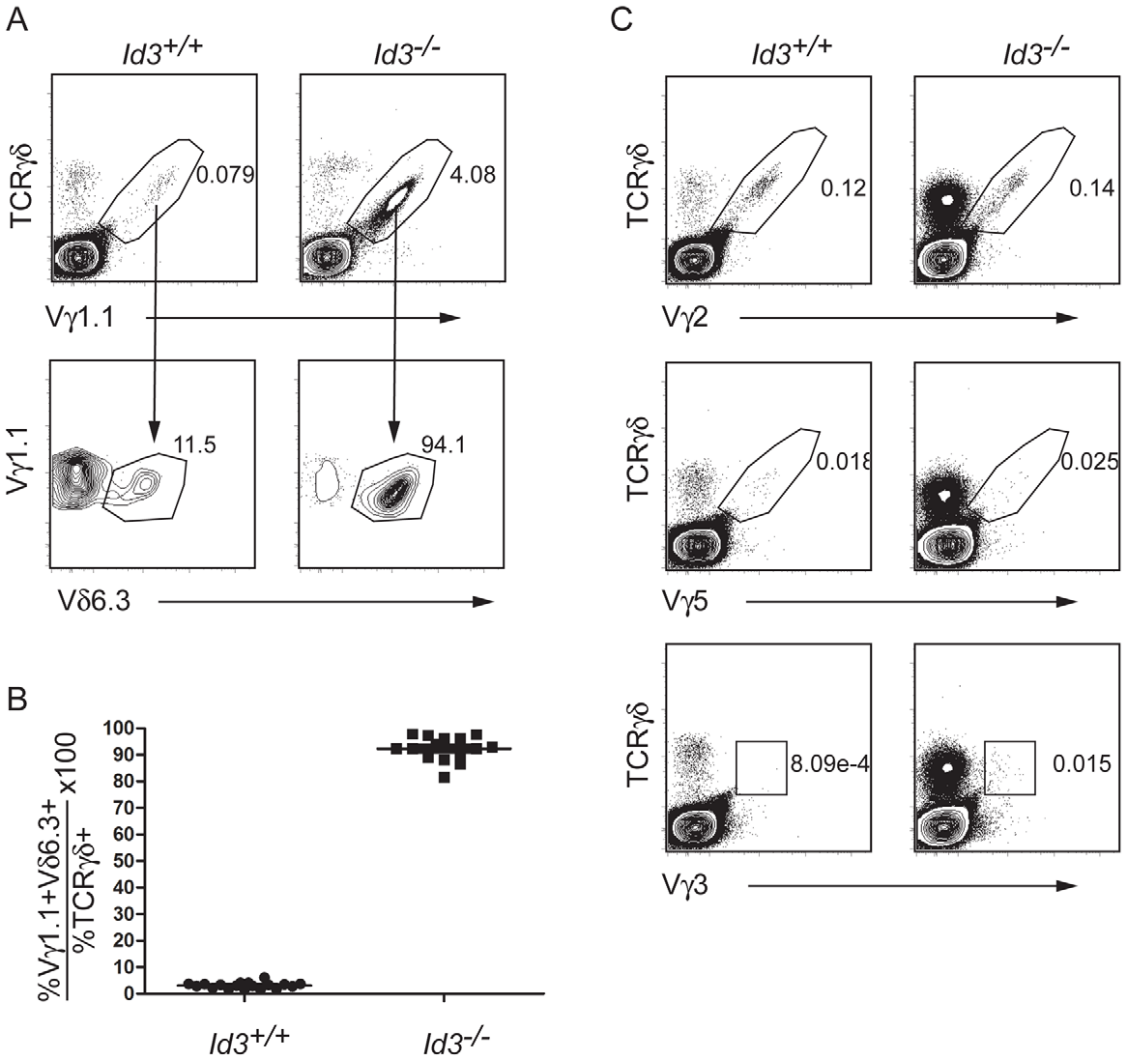
*Id3^−/−^* γδ T cells are highly enriched for cells with a Vγ1.1/Vδ6.3 TCR. A) FACS analysis of thymocytes for TCRγδ and Vγ1.1 (top panel). The frequency of TCRγδ^+^Vγ1.1^+^ cells is shown. TCRγδ^+^Vγ1.1^+^ cells were analyzed for expression of Vδ6.3 (bottom panel). The frequency of Vγ6.3^+^ cells in the TCRγδ^+^Vγ1.1^+^ population is shown. B) Frequency of Vγ1.1^+^Vδ6.3^+^ cells among TCRγδ^+^ cells in *Id3* ^+/+^ and *Id3* ^−/−^ mice. Data were calculated by dividing the frequency of Vγ1.1^+^Vδ6.3^+^ cells by the frequency of TCRγδ^+^ cells (×100). Each dot represents the frequency from on mouse. The line represents the average value. C) Analysis of thymocytes for expression of TCRγδ and Vγ2, Vγ5 or Vγ3 in *Id3^+/+^* and *Id3^−/−^* thymocytes. One of 4 representative experiments is shown.

The presence of a large population of activated γδ T cells in the *Id3* ^−/−^ neonatal thymus suggests that these cells derive from cells that underwent V(D)J recombination in the late embryonic or neonatal period. To gain insight into the origin of the majority of *Id3^−/−^* γδ T cells, we examined their TCR repertoire by staining with a panel of anti-Vγ antibodies. This analysis revealed that >90% of γδ T cells in the *Id3^−/−^* thymus express Vγ1.1 (**Fig. 4A, B**). This increase in Vγ1.1 usage is not at the expense of the other Vγ gene segments since the total number of Vγ2^+^ and Vγ5^+^ γδ T cells are similar to that in the *WT* thymus, although their frequency within the γδ T cell population is reduced (**Fig. 4C**).

Importantly, the majority of Vγ1.1^+^ cells in the *Id3^−/−^* thymus co-express Vδ6.3 (**Fig. 4A**). Vγ1.1^+^Vδ6.3^+^ γδ T cells have been reported to be of late fetal origin, express CD4 and have an activated phenotype including high expression of CD44, low expression of CD24 with rapid production of IFNγ and IL4 similar to what we have observed with *Id3^−/−^* γδ T cells [22]. These observations lead us to conclude that *Id3* deficiency allows for an increase in the number of Vγ1.1^+^Vδ6.3^+^ γδ T cells without a major effect on Vγ2^+^ or Vγ5^+^ γδ T cells.

### Limited Diversity in Vγ1.1-Jγ4 and Vδ6-Jδ1 Rearrangements in *Id3* ^−/−^ γδ T Cells

**Figure 5:**
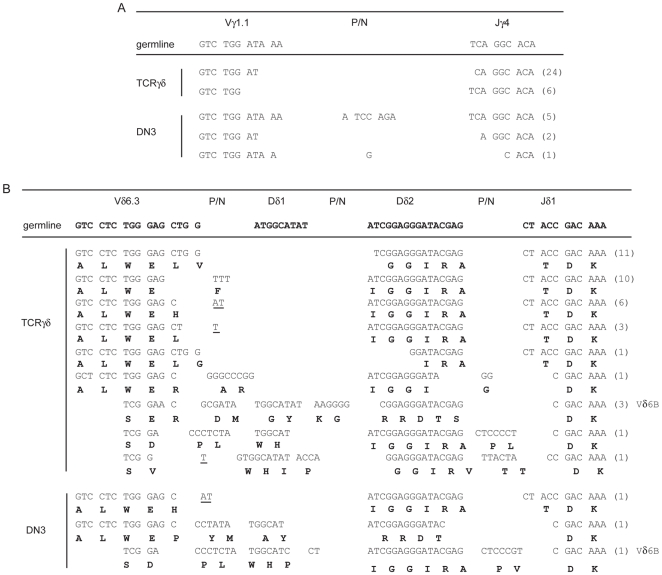
Sequence analysis of Vγ1.1-Jγ4 and Vδ6-Jδ1 junctions. Sequence of Vγ1.1 and Jγ4 (A) or Vδ6 and Jδ1 (B) junctions from TCRγδ^+^ T cells (upper, indicated on the right) or DN3 thymocytes (lower, indicated on the right). Data are cumulative from 2 independent experiments in which DNA was isolated 50,000 TCRγδ^+^ cells and 30,000 Lin^−^c-*kit* ^−^CD25^+^ (DN3) cells. In each experiment 3 independent PCR amplifications were performed on each population and cloned into pBSK for sequencing. A minimum of 30 sequences were analyzed for each population. Results from the two experiments were essentially identical. Only in-frame sequences are shown and the number of clones sharing the identical sequence is indicated on the right in parenthesis. The amino acid sequence is shown in bold below the DNA sequence. The Vδ6.3 primer also amplifies the Vδ6B gene segment and sequences derived from Vδ6B are indicated on the right. P/N represent potential P or N additions and the underlined sequences are potential P additions. Sequences derived from the Dδ1 and Dδ2 gene segments are also indicated.

In WT mice Vγ1.1^+^Vδ6.3^+^ T cells develop from fetal precursors that rearrange the γ and the δ chains in late embryonic life [23]. These cells show frequent rearrangement of the Vγ1.1 variable gene segment to the Jγ4 joining segment and of Vδ6.3 to Jδ1 and, depending on the genetic background of the mice, can have oligoclonal or polyclonal junctional sequences [45], [46]. To gain insight into the complexity of the rearrangements in *Id3^−/−^* Vγ1.1^+^Vδ6.3^+^ cells we amplified and sequenced the Vγ1.1-Jγ4 and Vd6-Jδ1 junctions in the TCRγ^+^ population. Analysis of Vγ1.1-Jγ4 junctions revealed that 30 of 31 sequences were in-frame and consisted of only two unique sequences indicating a population of Vγ1.1^+^ T cells lacking significant TCR diversity. In addition, these sequences lacked N nucleotide additions suggesting that the rearrangements occurred in the absence of TdT (**Fig. 5A**). Analysis of Vδ6-Jδ1 junctions also revealed a lack of diversity with 32 of 37 in-frame sequences containing the Vδ6.3 gene segment, consistent with our flow cytometry analysis (**Fig. 5B**). Moreover, 21 of the 32 Vδ6.3-Jδ1 junctions are represented by only two sequences. In the majority of sequences the Dδ2-Jδ1 and Vδ6-Dδ2 junctions resulted in maintenance of the germline sequence and the Dδ1 gene segment was not observed in these junctions (**Fig. 5B**). Of the 4 unique sequences that showed diversity following the Vδ6.3 gene segment at least 2 represent potential P rather than N nucleotide additions. Notably, the invariable Dδ2-Jδ1 junction forces a unique reading frame of the Dδ2 segment (V/IGGIRA), which contributes to the CDR3 domain [47], thus resulting in a highly invariant Vγ1.1^+^Vδ6.3^+^ TCR, at least for those cells using the Vγ1.1-Jγ4 and Vδ6-Jδ1 rearrangement. The presence of a highly invariant receptor on cells with an activated phenotype suggests that the Vγ1.1^+^Vδ6.3^+^ T cells are selected by a ligand present in the thymus.

To examine the possibility that the Vγ1.1^+^Vδ6.3^+^ T cells in *Id3^−/−^* mice arise as a consequence of preferential Vγ1.1 and Vδ6.3 rearrangement in adult thymocytes we analyzed the Vγ1.1-Jγ4 and Vδ6-Jδ1 junctions in unselected *Id3* ^−/−^ DN3 cells. This analysis revealed that the in-frame Vγ1.1-Jγ4 rearrangements (8/15) contained 3 unique sequences that were distinct from those amplified from *Id3* ^−/−^ γδ T cells (**Fig. 5A**). In addition, only 3 of 16 Vδ6-Jδ1 junctions were in-frame and each of these sequences was unique with one sequence containing the Vδ6B gene segment (**Fig. 5B**). Therefore, *Id3^−/−^* DN3 cells show no evidence of a preferential production of the Vγ1.1-Jγ4 or Vδ6.3-Jδ1 junctions used in the γδ T cells in *Id3* ^−/−^ mice. Further, if a small number of Vγ1.1^+^Vδ6.3^+^ γδ T cells with this rearrangement developed in the adult and expanded we would expect this population of γδ T cells to incorporate more BrdU than WT γδ T cells. However, multiple in vivo BrdU incorporation experiments failed to reveal an increase in proliferation of *Id3* ^−/−^ γδ T cells (**Fig. S5**) [35]. Taken together, our results indicate that in the absence of Id3 there is an elevated number of Vγ1.1^+^Vδ6.3^+^ γδ T cells that originate during late fetal or neonatal life. Consistent with this conclusion, reconstitution of *WT* or *Id3^−/−^* mice with adult *Id3^−/−^* bone marrow hematopoietic stem and progenitor cells largely fails to reconstitute this γδ T cell population (**Fig. S6**).

### Development of Activated γδ T Cells in *Id3^−/−^* Mice Requires SAP

**Figure 6:**
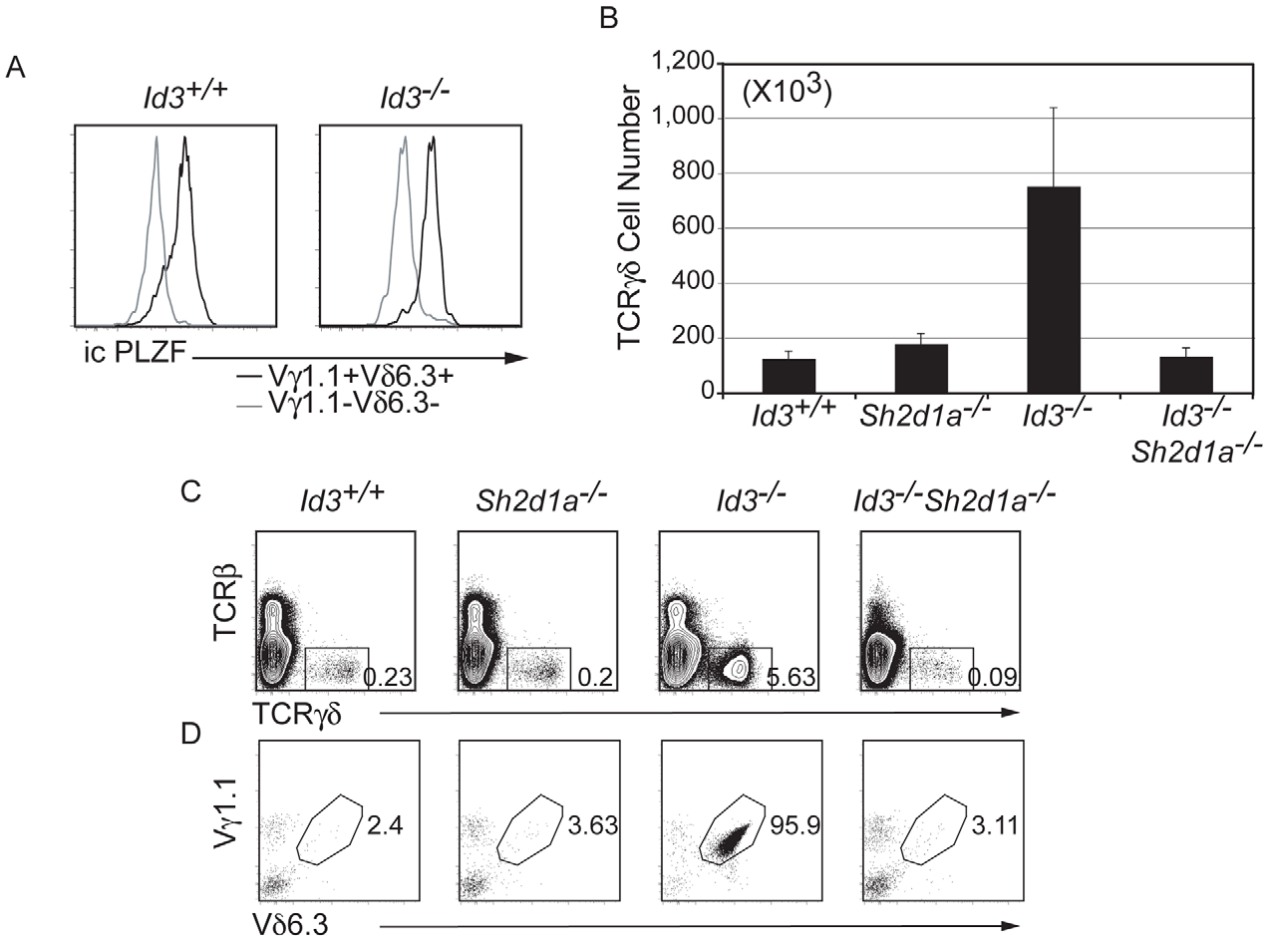
*Id3^−/−^* Vγ1.1^+^Vδ6.3^+^ T cells express PLZF and require SAP. A) Intracellular FACS analysis for expression of PLZF in *Id3* ^+/+^ and *Id3* ^−/−^ thymic Vγ1.1^+^Vδ6.3^+^ (black) and Vγ1.1^−^Vδ6.3^−^ (grey) γδ T cells. (B) Number of TCRγδ^+^ cells in the thymus of mice of the indicated genotype. *Sh2d1a* ^−/−^ mice fail to express the gene encoding SAP. Average +/− standard deviation derived from 6 mice. *Id3* ^−/−^ mice have more TCRγδ^+^ cells than any of the other genotypes (p<0.05) (C) FACS analysis for TCRβ and TCRγ expression on total thymocytes from mice of the indicated genotype. The frequency of TCRγδ^+^ cells is shown. (D) Analysis of TCRγδ^+^ cells for expression of Vγ1.1 and Vδ6.3. The frequency of Vγ1.1^+^Vδ6.3^+^ cells among TCRγδ^+^ cells is shown.

A subset of Vγ1.1^+^Vδ6.3^+^, referred to as γδ NKT, share phenotypic and functional characteristics with NKT cells including expression of the transcription factor PLZF and a requirement for SAP-dependent [24], [37], [39], [48]. However, some Vγ1.1^+^Vδ6.3^+^ T cells develop independent of SAP signaling [24]. To further establish the parallels between *Id3* ^−/−^ Vγ1.1^+^Vδ6.3^+^ cells and NKT cells we investigated the expression of PLZF. Importantly, PLZF was highly expressed in these cells compare to Vγ1.1^−^Vδ6.3^−^ γδ T cells, in both the *Id3^+/+^* and the *Id3^−/−^* thymus (**Fig. 6A**). We examined whether development of this activated γδ T cell population in *Id3* ^−/−^ mice requires SAP by generating *Id3^−/−^Sh2d1a^−/−^* mice. Strikingly, the total number of γδ T cells in *Id3^−/−^Sh2d1a^−/−^* mice was similar to that in *WT* and *Sh2d1a^−/−^* mice (**Fig. 6B and C**). Moreover, the frequency of Vγ1.1^+^Vδ6.3^+^ γδ T cells in *Id3^−/−^Sh2d1a^−/−^* mice was similar to *WT* and *Id3^−/−^Sh2d1a^−/−^* γδ T cells showed no evidence of an activated phenotype (**Fig. 6D** **and Fig. S7**). Therefore, activation of the SAP signaling pathway is essential for the γδ T cell phenotype observed in *Id3^−/−^* mice. These data indicate that SAP is essential for development or survival of the Vγ1.1^+^Vδ6.3^+^ T cells present in *Id3* ^−/−^ mice. Interestingly, all of the observed alterations in the *Id3* ^−/−^ thymus were normalized by deletion of *Sh2d1a*. That is, γδ T cell numbers and phenotype as well as total thymocytes numbers are similar to *WT* in *Id3* ^−/−^ *Sh2d1a* ^−/−^ mice (**Fig. S7**). This finding is striking because deletion of γδ T cells in *Id3* ^−/−^ mice, by creating *Id3* ^−/−^ *Tcrd* ^−/−^ mice, does not restore thymic cellularity to *WT* levels (**Fig. S8**). Therefore, multiple alterations in the *Id3* ^−/−^ thymus are dependent on SAP signaling.

### Development of Activated γδ T Cells in *Id3^−/−^* Mice Requires *E2A*

**Figure 7:**
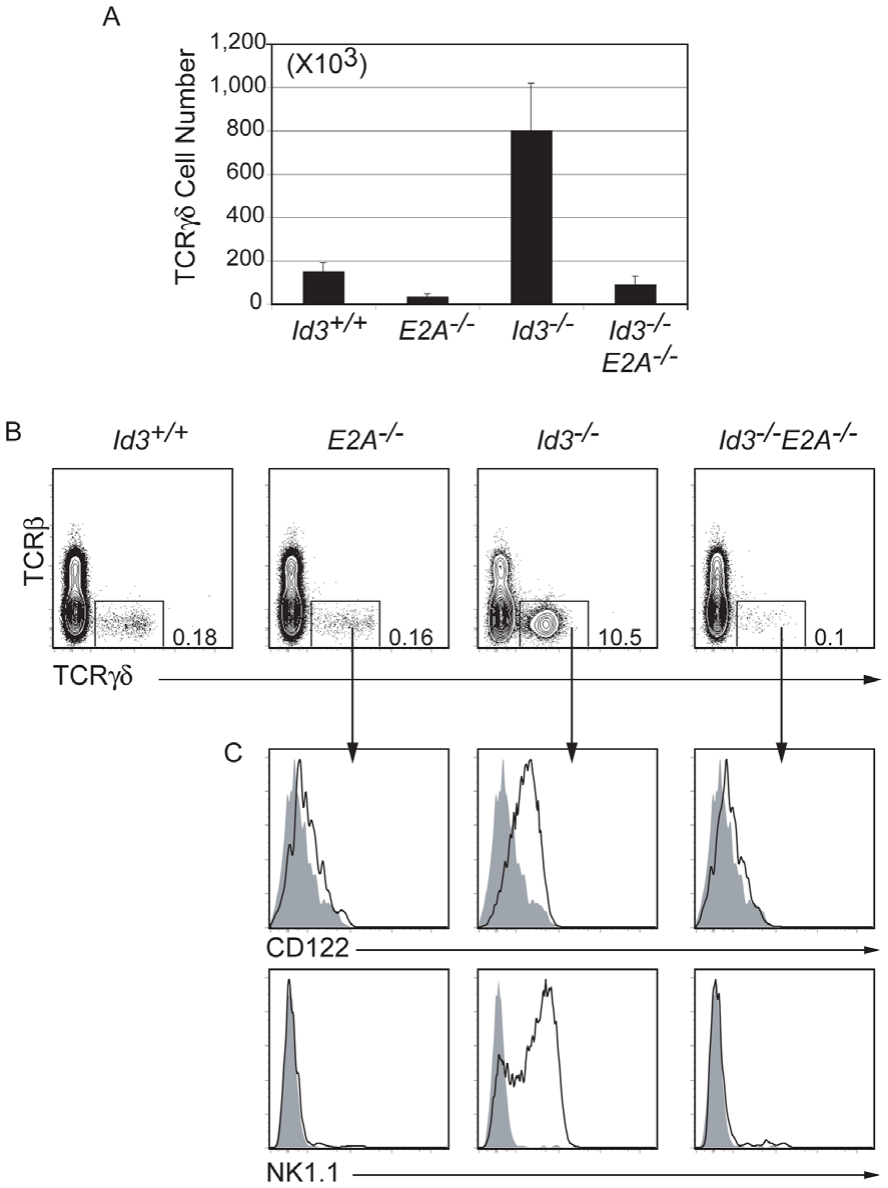
γδ T cell development in *Id3^−/−^* mice is E2A dependent. A) Total number of TCRγδ^+^ cells in the thymus of *Id3^+/+^*, *E2A^−/−^*, *Id3^−/−^ and Id3^−/−^E47^−/−^* mice. Bars represent the average +/− standard deviation. p<0.001 for *Id3* ^−/−^ compared to all other genotypes. B) Flow cytometric analysis for TCRβ and TCRγδ in mice with the indicated genotype. The frequency of TCRγδ^+^ cells is shown. (C) Analysis of TCRγδ^+^ cells for CD122 (upper plots) and NK1.1 (lower plots) expression. Genotypes are as indicated in (C) (for the open histogram) and are shown relative to *Id3* ^+/+^ TCRγδ^+^ cells (shaded histogram), which served as a negative control. Results are representative from at least 6 mice of each genotype.

Id3 is a transcriptional repressor that prevents E proteins from binding DNA [49]. All of the E proteins are expressed in T cells; however, deletion of E2A is sufficient to restore αβ T cell maturation defects in *Id3^−/−^* [50]. Therefore, we tested the requirement for E2A in the development of activated γδ T cells in *Id3* ^−/−^ mice by generating *Id3^−/−^E2A^−/−^* mice. Consistent a previous study we found that *E2A* is required for development of normal numbers of γδ T cells (**Fig. 7A and B**) [51]. Importantly, mice that lack both *Id3* and *E2A* have fewer γδ T cells than *WT* mice but more γδ T cells than *E2A* ^−/−^ mice **(** **Fig. 7A and B** **)**. Nonetheless, the γδ T cells that develop in *Id3* ^−/−^ *E2A* ^−/−^ mice fail to express CD122 and NK1.1 (**Fig. 7C** **)** Therefore, E2A is required for the development of γδ T cells with an activated phenotype in *Id3* ^−/−^ mice.

## Discussion

In this study, we report that *Id3*-deficiency results in a 8-fold increase in the number of γδ T cells in the thymus and that the majority of these cells likely express an invariant Vγ1.1^+^Vδ6.3^+^ TCR. Similar to WT Vγ1.1^+^Vδ6.3^+^ cells, *Id3* ^−/−^ γδ T cells have high expression of CD122, CD44 and NK1.1, low expression of CD24, and rapidly secrete IFNγ and IL4 after *in vitro* stimulation. The “activated” phenotype of these γδ T cells parallels that of NKT cells, a finding that has led to the hypothesis that Vγ1.1^+^Vδ6.3^+^ T cells represent an innate branch within the γδ T cell lineage [25]. Here, we demonstrate that both *Id3* ^+/+^ and *Id3^−/−^* Vγ1.1^+^Vδ6.3^+^ cells express the transcription factor PLZF, a molecular determinant of the NKT cell fate [37]. Moreover, we find that SAP is essential for development of *Id3* ^−/−^ Vγ1.1^+^Vδ6.3^+^ T cells, as is the case for NKT cells [39], [52], [53]. We, and others, have found that the majority of adult γδ T cells in *Id3* ^−/−^ mice proliferate at a rate similar to WT γδ T cells indicating that the *Id3* ^−/−^ Vγ1.1^+^Vδ6.3^+^ population is not increased because of extensive proliferation in the adult thymus [35], rather, we conclude that these γδ T cells expand during neonatal life. Our data are consistent with a model in which Id3 controls the response of Vγ1.1^+^Vδ6.3^+^ T cells to ligand- and/or SAP-mediated proliferation.

We demonstrate that the increased number of γδ T cells in *Id3* ^−/−^ mice is attributed to the increase in SAP-dependent cells. Deletion of *Sh2d1a* in *Id3* ^−/−^ mice abrogated the increase in γδ T cell numbers and the activated phenotype. Therefore, the major effect of *Id3*-deficiency on γδ T cell development is an increase in embryonically derived Vγ1.1^+^Vδ6.3^+^ T cells. This conclusion is in contrast to a previous report suggesting that alterations in adult DN3 cells underlie the increased production of γδ T cells in *Id3* ^−/−^ mice [35]. This conclusion was based, in part, on the observation that *Id3* ^−/−^ γδ T cells have less germline DNA at the TCRβ locus than WT γδ T cells. This finding led the authors to conclude that the γδ T cells developing in *Id3* ^−/−^ mice derive from cells that have an extended opportunity for TCRβ rearrangement. Our findings suggest that the reason for the increased TCRβ rearrangement may stem from differences in fetal versus adult cells rather than differences in *Id3* ^+/+^ and *Id3* ^−/−^ adult DN3 cells. Our findings are also inconsistent with a model in which Id3 plays a critical role in selection of self-ligand reactive γδ T cells; however, many of the *Id3* ^−/−^ Vγ1.1^+^Vδ6.3^+^ cells express CD4 or CD8 which is consistent with a failure to prevent some aspects of αβ T cell development [54]. More importantly, our data reveal that SAP-dependent signaling pathways are critically linked to the altered phenotype of *Id3* ^−/−^ T cells since the thymus of *Id3* ^−/−^ *Sh2d1a* ^−/−^ mice, unlike the *Id3* ^−/−^ or the *Id3* ^−/−^ *Tcrd* ^−/−^ thymus, is indistinguishable from the *Id3^+/+^* or *Shld1a* ^−/−^ thymus with respect to cellularity and phenotype.

Our hypothesis that Id3 functions downstream of TCR signals to limit SAP-dependent proliferation in γδ T cells is consistent with previous studies demonstrating that *Id3* is a target of TCR triggered signaling in both αβ and γδ T cells [30], [32]. The pathway from the TCR leading to *Id3* involves the MAP kinases Erk1 or Erk2, which are triggered by the Tec kinases *Itk* and *Rlk* [55]. Recently, *Itk* ^−/−^ mice were reported to have an increased number of PLZF-expressing Vγ1.1^+^Vδ6.3^+^ T cells, implying that *Itk* may also limit development of “innate” γδ T cells [25], [56]. Our data are consistent with the hypothesis that Id3 is an essential effector of the TCR-Itk-MAP kinase pathway that determines the consequence of signaling through the Vγ1.1^+^Vδ6.3^+^ TCR.

Id3 is an inhibitor of E protein DNA binding [57]. We, and others, found that deletion of *E2A* in *Id3* ^−/−^ mice blunted the development of activated γδ T cells indicating that elevated E2A function is critical for development of these cells [35]. It should be noted that E2A is required for normal γδ T cells development and affects the timing of rearrangement of specific Vγ receptors [21], [51]. Therefore, loss of activated γδ T cells in *E2A* ^−/−^ *Id3* ^−/−^ mice, as compared to *Id3* ^−/−^ mice, could be the result of E2A functions upstream of TCR signaling and independent of Id3. However, it seems likely that *Id3* deletion leads to heightened E2A (or E protein) activity after TCR-initiated signaling events, where E2A activity would normally be inhibited. In the case of γδ T cells, elevated activity of E2A may cooperate with SAP-dependent signals to promote an outcome from TCR-mediated signaling that is not typical, for example, leading to prolonged survival or proliferation.

Our analysis of Vγ1.1-Jγ4 and Vδ6-Jδ1 sequences in *Id3* ^−/−^ γδ T cells and DN3 thymocytes lead us to conclude that the majority of Vγ1.1^+^Vδ6.3^+^ cells in *Id3* ^−/−^ mice develop during fetal or neonatal life. This analysis revealed that *Id3* ^−/−^ γδ T cells have germline sequences at the Vγ1.1-Jγ4 and Dδ2-Jδ1 junctions, very low diversity in the Vδ6.3-Dδ2 junction and complete absence of the Dδ1 segment. However, Vγ1.1-Jγ4 and Vδ6-Jδ1 sequences retrieved from *Id3* ^−/−^ DN3 progenitors are characterized by diverse junctions. It is possible that the Vγ1.1-Jγ4 and Vδ6-Jδ1 sequences observed in *Id3* ^−/−^ γδ T cells could be generated from adult DN3 cells and that thymic selection leads to expansion of these cells. However, two observations argue against this possibility. First, *Id3* ^−/−^ γδ T cells proliferate to a similar extent as *WT* γδ T cells in the adult thymus and second, development of Vγ1.1^+^Vδ6.3^+^ T cells is blunted in WT or *Id3* ^−/−^ mice reconstituted with *Id3* ^−/−^ adult bone marrow. Therefore, adult thymic progenitors do not efficiently recapitulate the γδ T cell phenotype observed in *Id3* ^−/−^ mice. The activated phenotype of *Id3* ^−/−^ Vγ1.1^+^Vδ6.3^+^ T cells is consistent with the hypothesis that this receptor recognizes a ligand in the thymus. γδ T cells that recognize the unconventional MHC molecule T10- or T22 and Vγ3^+^Vδ1^+^ DETCs, which are also hypothesized to be ligand-selected have a similar phenotype [58].

Our results reveal an important role for Id3 in limiting the number of Vγ1.1^+^Vδ6.3^+^ T cells. Since Vγ1.1^+^Vδ6.3^+^ T cells share many features with NKT cells including rapid production of IFNγ and IL-4, their increased numbers could significantly alter immune responses. Indeed, *Itk* ^−/−^ mice, which also have an increased number of Vγ1.1^+^Vδ6.3^+^ T cells, have elevated serum IgE that is dependent on γδ T cells [25], [56]. Therefore, while Id3 appears to be largely dispensable for development of conventional γδ T cells, it limits the number of PLZF-expressing SAP-dependent “innate” γδ T cells.

## Materials and Methods

### Ethics Statement

All animal experiments were performed in compliance with the requirements of the University of Chicago Institutional Animal Care and Use Committee

### Mice

Mice were housed at The University of Chicago Animal Resource Center. *Id3* ^−/−^ and *Tcrd* ^−/−^ mice were purchased from Jackson ImmunoResearch. *Sh2d1a* ^−/−^ mice were a kind gift from C. Terhorst. *Genotyping was as previously described* [59], [60], [61]. All experiments were performed on mice that were 6 to 8 weeks old unless otherwise indicated.

### Antibodies, Flow Cytometry and Cell Sorting

Cells were blocked with anti-FcγR prior to staining with specific antibodies conjugated to biotin, FITC, PE, PE-Cy7 or APC, acquired in a FACS Canto using FACSDiva software and analyzed with FLOWjo. In all experiments viable cells were gated based on forward and side scatter profiles and dead cells were further excluded using Propidium iodide. Sorting was performed on a FACSAria. The following antibodies were purchased from BD Biosciences or eBiosciences: CD4, CD8a, CD8b, TCRβ, TCRγδ, NK1.1, CD122, CD44, CD24, IFNγ, Vγ2, Vγ3, ckit, and CD25. Anti-Vγ1.1 (2.11), -Vγ5 (F2.67) and -Vδ6.3 (9D3) were described previously [45]. For analysis of Vγ1.1^+^Vδ6.3^+^ cells a minimum of 2,000 TCRγδ ^+^ cells were analyzed in each experiment.

### Cytometric Bead Assay (CBA) and IFNγ Assay

Phorbol 12-myristate 13-acetate (PMA) plus ionomycin treatment and intracellular staining for IFNγ were described previously [44]. CBA analysis was as described in [62].

### Real-Time Quantitative (Q)PCR

RNA from TCRβ^+^CD4 and TCRβ^+^CD8 thymocytes was DNAase-treated and reverse-transcribed using Superscript III (Invitrogen). QPCR was performed with gene-specific primers in a iCycler (BioRad), using the iQ SYBR Green Supermix (BioRad). A standard curve was included for each primer set. *Sox13* primers: qSox13(3047)for: 5′-CCCTATTTCTCTCCAGACTGT-3′ and qSox13(3142)rev: 5′-CTGGTTAAGTTATTCATCATTATC-3′. Hprt primers were reported previously [63].

### Cloning and Sequencing

DNA was isolated from 50,000 TCRγδ^+^ or 30,000 Lin-c*kit*-CD25^+^ thymocytes as previously described [51]. Vγ1.1-Jγ4 and Vδ6-Jδ1 rearrangements were then amplified by the following primers: Vγ1.1 for: 5′-CCGGCAAAAAGCAAAAAAGTT-3′ and Jγ4 rev: 5′-GCAAATATCTTGACCCATGA-3′, Vδ6uni for: 5′-AYTCTGTAGTCTTCCAGAAATCA-3′ and Jδ1 rev: 5′- TTGGTTCCACAGTCACTTGG-3′. PCR products were ligated to pGEM-T vector (Promega). DNA from single colonies was extracted and sequenced at the University of Chicago Cancer Research Center Sequencing Facility using the T7 primer. Three independent amplifications were performed from each sample in 2 independent experiments.

## Supporting Information

Figure S1 *Id3^−/−^* mice have 3-fold fewer thymocytes than *Id3^+/+^* mice. Total number of thymocytes in *Id3^+/+^* and *Id3^−/−^* mice. Bars represent the average ± standard deviation from at least 10 mice. p<0.0005.
(2.54 MB TIF)

Figure S2 *Id3^−/−^* mice have an increased number of γδ T cells in the spleen that express CD4 and CD8. A) Flow cytometric analysis of *Id3^+/+^* and *Id3^−/−^* splenocytes for TCRβ and TCRγδ. Total splenocytes were first gated for viable cells using propidium idodie (PI). B) Total number of TCRγδ^+^ cells in the spleen of *Id3^+/+^* and *Id3^−/−^* mice. Bars represent the average ± standard deviation from >15 mice. p<0.001. C) TCRγδ^+^ cells were analyzed for CD4 and CD8 expression. D) Total number of CD4+, CD8+, DN and DP splenocytes expressing TCRγδ in the spleen *Id3^+/+^* (grey) and *Id3^−/−^* (black) mice. Bars represent the average ± standard deviation from >15 mice. p<0.001 for all *Id3^+/+^* to *Id3^−/−^* comparisons. E) Flow cytometric analysis of *Id3^−/−^* CD8α+ splenocytes analyzed for expression of TCRβ (left panel) or TCRγδ (right panel) and CD8β.
(10.44 MB TIF)

Figure S3 *Id3^−/−^* γδ splenocytes have characteristics of activated cells. Flow cytometric analysis of *Id3^+/+^* DN splenocytes (A) or *Id3^−/−^* DN, CD4 or CD8 splenocytes (B) for expression of CD122, CD44, CD24 or NK1.1. Data are representative of more than 10 independent experiments. C) Flow cytometric analysis showing intracellular IFNγ expression in *Id3^+/+^* and *Id3^−/−^* TCRγδ^+^ splenocytes 5 hours after stimulation with PMA and ionomycin. The shaded histogram shows staining with an isotype control antibody, open histogram shows staining with anti-IFNg antibody. One of 3 independent experiments is shown.
(9.53 MB TIF)

Figure S4 *Id3^−/−^* γδ T cells make IFNγ and IL-4 after *in vitro* stimulation. (A) Total thymocytes from *Id3^+/+^* or *Id3^−/−^* mice were cultured *in vitro* with (lower panels) or without (upper panels) PMA plus ionomycin for 5 hours. Intracellular staining for IFNγ and IL-4 on TCRγδ^+^ cells is shown. The frequency of cells producing both IFNγ and IL-4 is indicated. (B) Cytometric bead assay for IFNγ, IL4, IL10 and IL13 produced from anti-CD19, anti-TCRβ and anti-Ter119 depleted splenocytes 72 hours after stimulation with anti-TCRγ antibody. PMA+ionomycin stimulated thymocytes from *Id3^−/−^* mice are shown as a positive control.
(10.12 MB TIF)

Figure S5 *Id3^−/−^* γδ T cells do not hyper-proliferate. BrdU incorporation in TCRγδ^+^ thymocytes (upper panels) and splenocytes (lower panels) from *Id3^+/+^* (left panels) and *Id3^−/−^* (right panels) mice 16 hours after BrdU injection.
(4.95 MB TIF)

Figure S6 *Id3^−/−^* adult hematopoietic progenitors fail to reconstitute the γδ T cell phenotype in *Id3^+/+^* or *Id3^−/−^* mice. (A) Total bone marrow cells from *Id3^+/+^* and *Id3^−/−^* (Ly5.2+) mice were injected into lethally irradiated (1000 rad) *Id3^+/+^* and *Id3^−/−^* Ly5.1+ mice and thymocytes were analyzed 6 or 12 weeks post-reconstitution. Flow cytometric analysis for TCRβ and TCRγδ on total thymocytes is shown. Plots are representative of 2–3 independent experiments B) TCRβ versus TCRγδ profile for *Id3^−/−^* thymus for comparison. (C) Total numbers of γδ T cells in *Id3^+/+^* and *Id3^−/−^* mice without reconstitution (none) or after reconstitution in *Id3^+/+^* or *Id3^−/−^* hosts. The average ± standard deviation from 3 independent experiments is shown.
(7.75 MB TIF)

Figure S7 Deletion of *Sh2d1a* restores thymus cellularity and reverses the activated phenotype of γδ cells in *Id3^−/−^* mice. (A) Total thymocytes numbers in mice of the indicated genotypes. At least 4 mice were analyzed for each genotype. p<0.01 for *Id3^−/−^Sh2d1a^+/+^* compared to *Id3^−/−^Sh2d1a^−/−^* or *Id3^+/+^Sh2d1a^−/−^*. (B) CD122, CD44, CD24 and NK1.1 expression on mice of the indicated genotype (black histogram) compared to *Id3^+/+^* γδ cells (grey histogram). Results are representative from more than 6 mice for each genotype.
(9.63 MB TIF)

Figure S8 Deletion of *Tcrd* does not restore thymus cellularity in *Id3^−/−^* mice. (A) Total thymocytes numbers in mice of the indicated genotypes. At least 4 mice were analyzed for each genotype. p<0.01 for *Id3^+/+^Sh2d1a^−/−^* or *Id3^−/−^Tcrd^−/−^* compared to *Id3^+/+^Tcrd^−/−^*.
(3.93 MB TIF)
