# Supplementary figures and images for: Inhibitor of DNA Binding 3 Limits Development of Murine Slam-Associated Adaptor Protein-Dependent “Innate” γδ T cells
Source: PLoS One. 2010 Feb 19;5(2):e9303. doi: 10.1371/journal.pone.0009303 (PMC2824806; doi:10.1371/journal.pone.0009303)

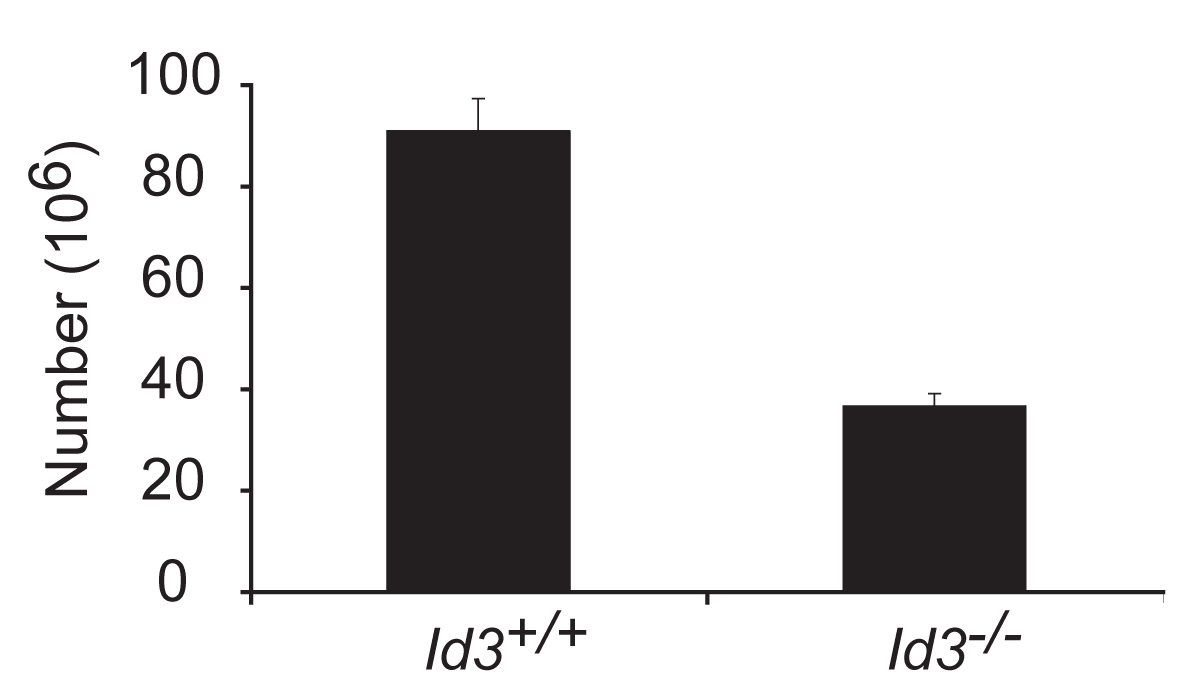

Supplement: Figure S1 — Id3−/− mice have 3-fold fewer thymocytes than Id3+/+ mice. Total number of thymocytes in Id3+/+ and Id3−/− mice. Bars represent the average ± standard deviation from at least 10 mice. p<0.0005. (2.54 MB TIF) [file pone.0009303.s001.tif]

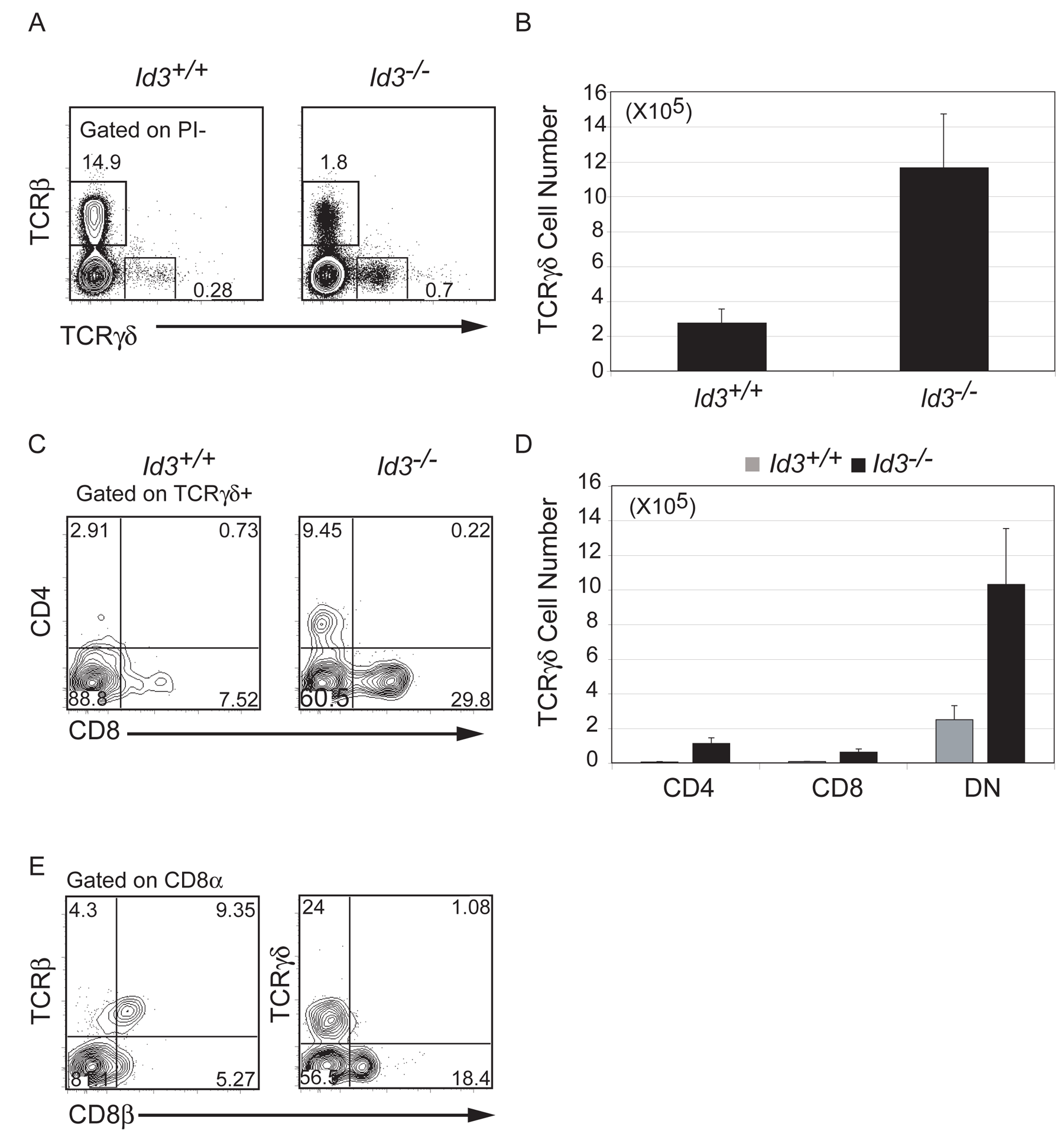

Supplement: Figure S2 — Id3−/− mice have an increased number of γδ T cells in the spleen that express CD4 and CD8. A) Flow cytometric analysis of Id3+/+ and Id3−/− splenocytes for TCRβ and TCRγδ. Total splenocytes were first gated for viable cells using propidium idodie (PI). B) Total number of TCRγδ+ cells in the spleen of Id3+/+ and Id3−/− mice. Bars represent the average ± standard deviation from >15 mice. p<0.001. C) TCRγδ+ cells were analyzed for CD4 and CD8 expression. D) Total number of CD4+, CD8+, DN and DP splenocytes expressing TCRγδ in the spleen Id3+/+ (grey) and Id3−/− (black) mice. Bars represent the average ± standard deviation from >15 mice. p<0.001 for all Id3+/+ to Id3−/− comparisons. E) Flow cytometric analysis of Id3−/− CD8α+ splenocytes analyzed for expression of TCRβ (left panel) or TCRγδ (right panel) and CD8β. (10.44 MB TIF) [file pone.0009303.s002.tif]

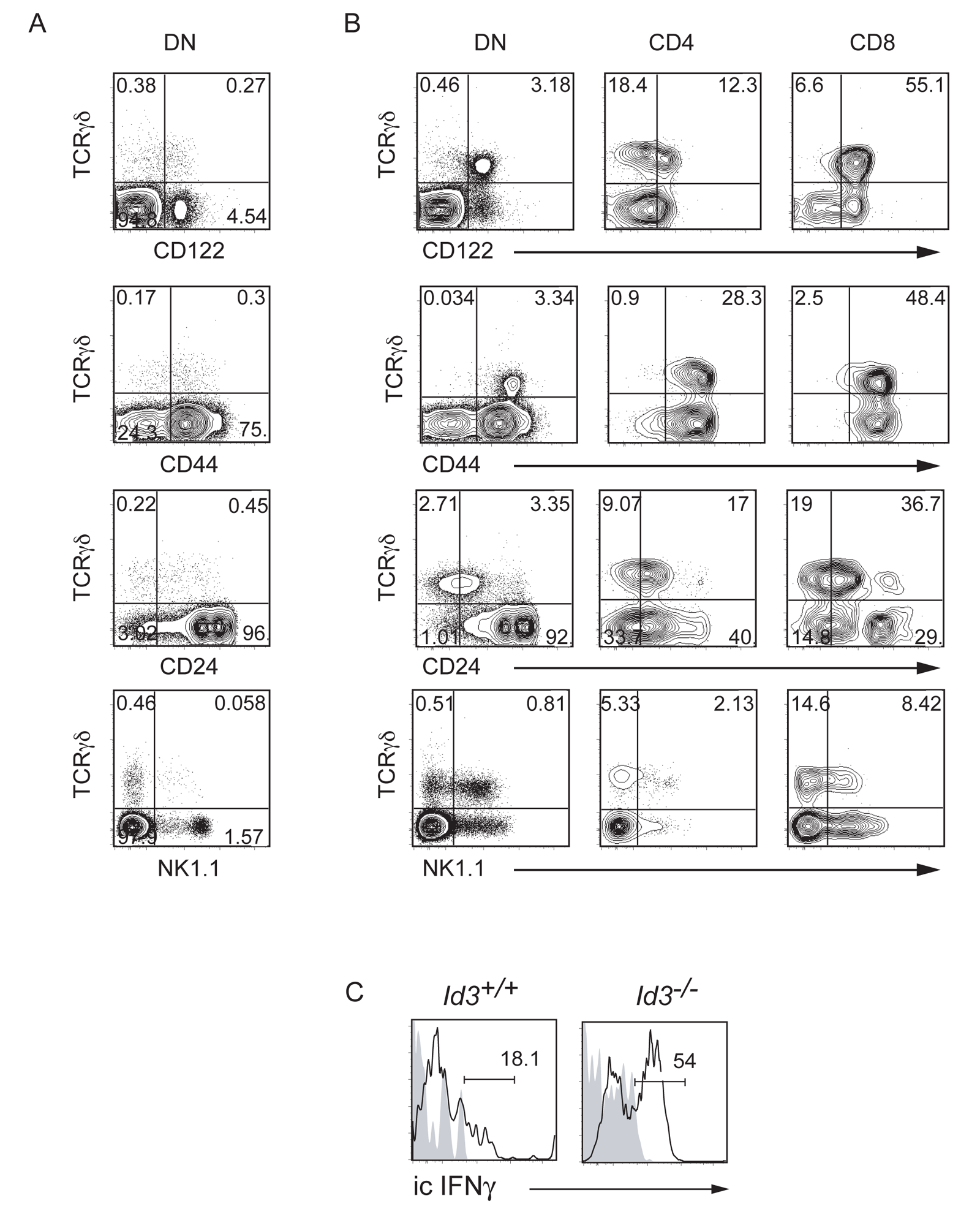

Supplement: Figure S3 — Id3−/− γδ splenocytes have characteristics of activated cells. Flow cytometric analysis of Id3+/+ DN splenocytes (A) or Id3−/− DN, CD4 or CD8 splenocytes (B) for expression of CD122, CD44, CD24 or NK1.1. Data are representative of more than 10 independent experiments. C) Flow cytometric analysis showing intracellular IFNγ expression in Id3+/+ and Id3−/− TCRγδ+ splenocytes 5 hours after stimulation with PMA and ionomycin. The shaded histogram shows staining with an isotype control antibody, open histogram shows staining with anti-IFNg antibody. One of 3 independent experiments is shown. (9.53 MB TIF) [file pone.0009303.s003.tif]

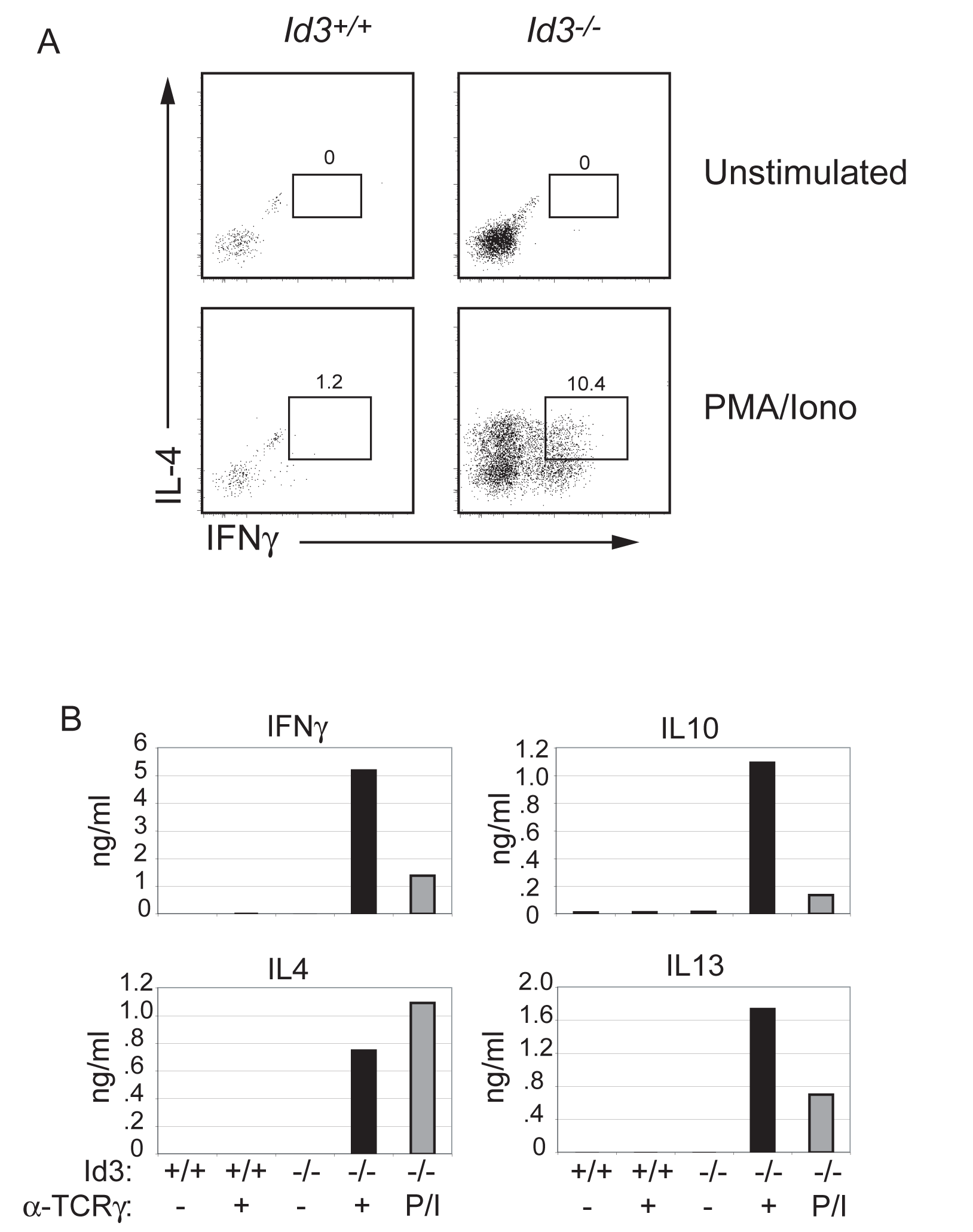

Supplement: Figure S4 — Id3−/− γδ T cells make IFNγ and IL-4 after in vitro stimulation. (A) Total thymocytes from Id3+/+ or Id3−/− mice were cultured in vitro with (lower panels) or without (upper panels) PMA plus ionomycin for 5 hours. Intracellular staining for IFNγ and IL-4 on TCRγδ+ cells is shown. The frequency of cells producing both IFNγ and IL-4 is indicated. (B) Cytometric bead assay for IFNγ, IL4, IL10 and IL13 produced from anti-CD19, anti-TCRβ and anti-Ter119 depleted splenocytes 72 hours after stimulation with anti-TCRγ antibody. PMA+ionomycin stimulated thymocytes from Id3−/− mice are shown as a positive control. (10.12 MB TIF) [file pone.0009303.s004.tif]

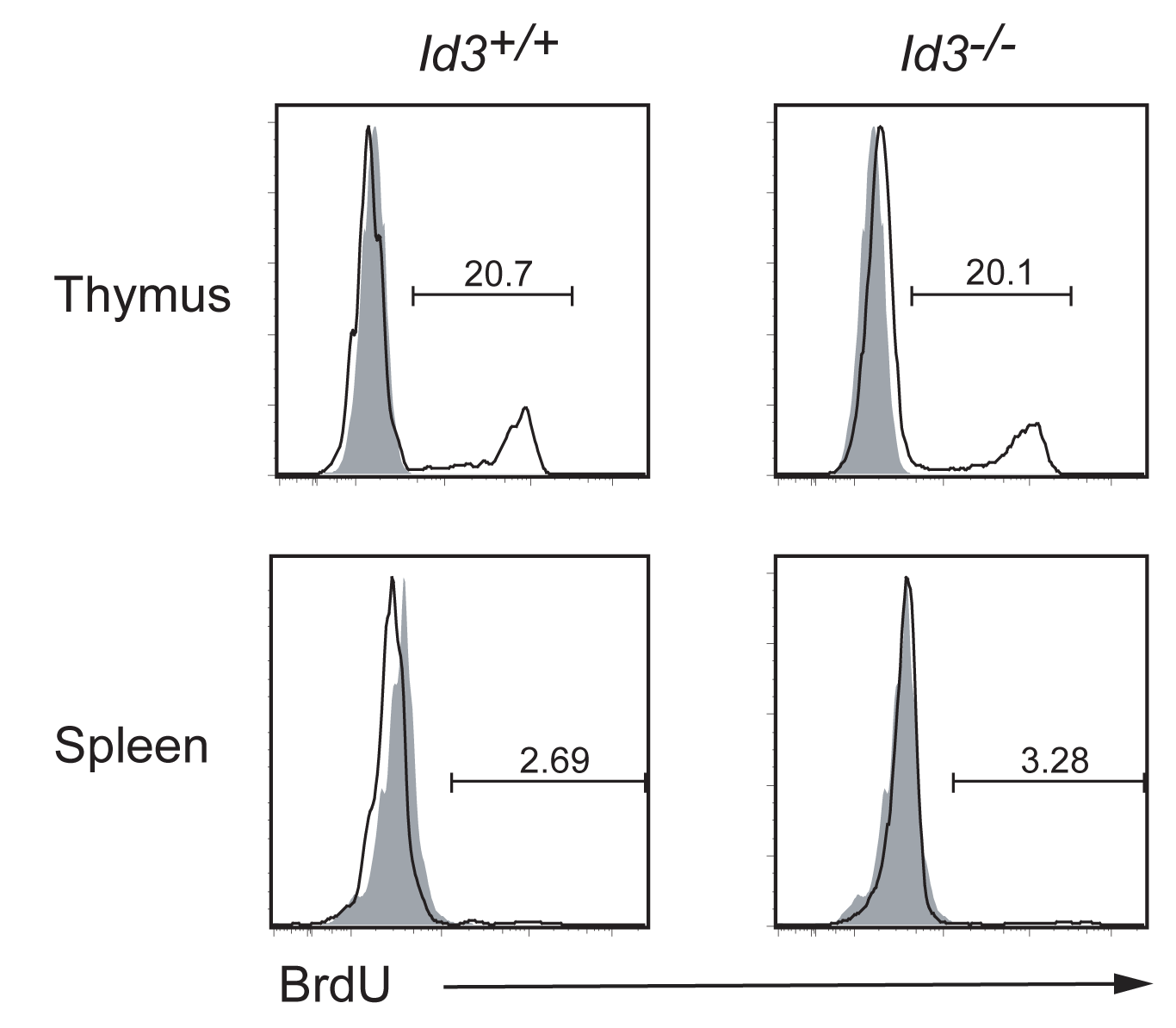

Supplement: Figure S5 — Id3−/− γδ T cells do not hyper-proliferate. BrdU incorporation in TCRγδ+ thymocytes (upper panels) and splenocytes (lower panels) from Id3+/+ (left panels) and Id3−/− (right panels) mice 16 hours after BrdU injection. (4.95 MB TIF) [file pone.0009303.s005.tif]

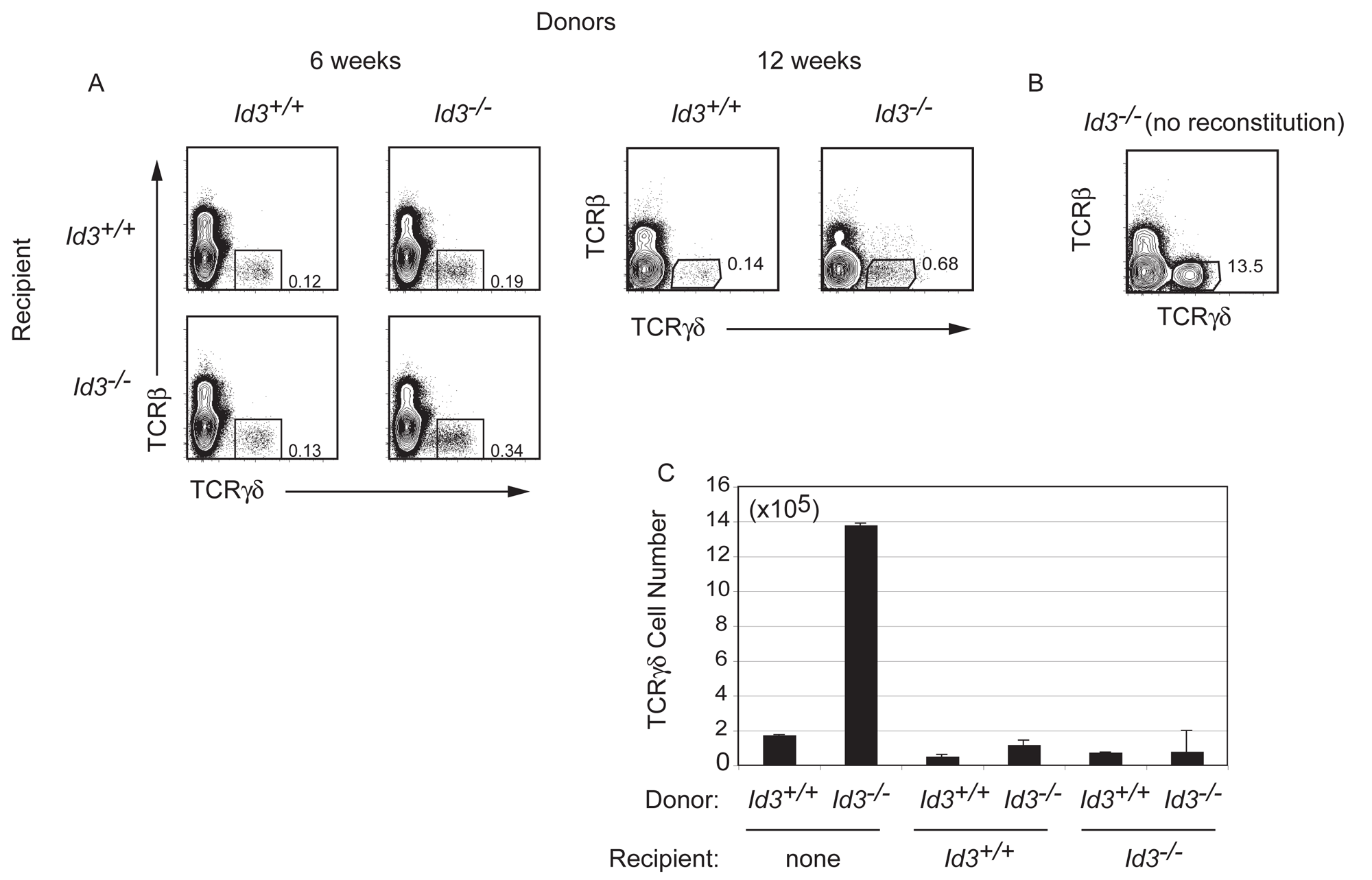

Supplement: Figure S6 — Id3−/− adult hematopoietic progenitors fail to reconstitute the γδ T cell phenotype in Id3+/+ or Id3−/− mice. (A) Total bone marrow cells from Id3+/+ and Id3−/− (Ly5.2+) mice were injected into lethally irradiated (1000 rad) Id3+/+ and Id3−/− Ly5.1+ mice and thymocytes were analyzed 6 or 12 weeks post-reconstitution. Flow cytometric analysis for TCRβ and TCRγδ on total thymocytes is shown. Plots are representative of 2–3 independent experiments B) TCRβ versus TCRγδ profile for Id3−/− thymus for comparison. (C) Total numbers of γδ T cells in Id3+/+ and Id3−/− mice without reconstitution (none) or after reconstitution in Id3+/+ or Id3−/− hosts. The average ± standard deviation from 3 independent experiments is shown. (7.75 MB TIF) [file pone.0009303.s006.tif]

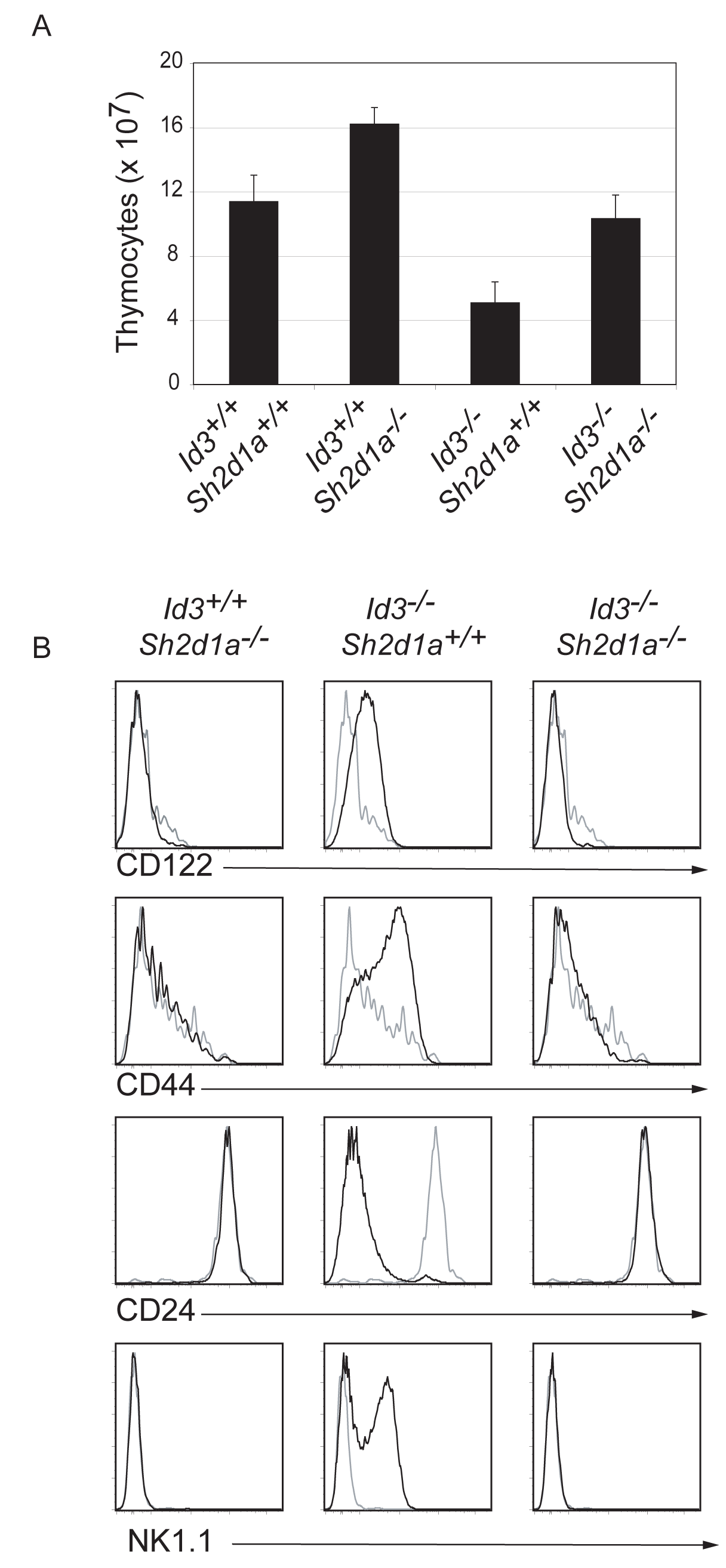

Supplement: Figure S7 — Deletion of Sh2d1a restores thymus cellularity and reverses the activated phenotype of γδ cells in Id3−/− mice. (A) Total thymocytes numbers in mice of the indicated genotypes. At least 4 mice were analyzed for each genotype. p<0.01 for Id3−/−Sh2d1a+/+ compared to Id3−/−Sh2d1a−/− or Id3+/+Sh2d1a−/−. (B) CD122, CD44, CD24 and NK1.1 expression on mice of the indicated genotype (black histogram) compared to Id3+/+ γδ cells (grey histogram). Results are representative from more than 6 mice for each genotype. (9.63 MB TIF) [file pone.0009303.s007.tif]

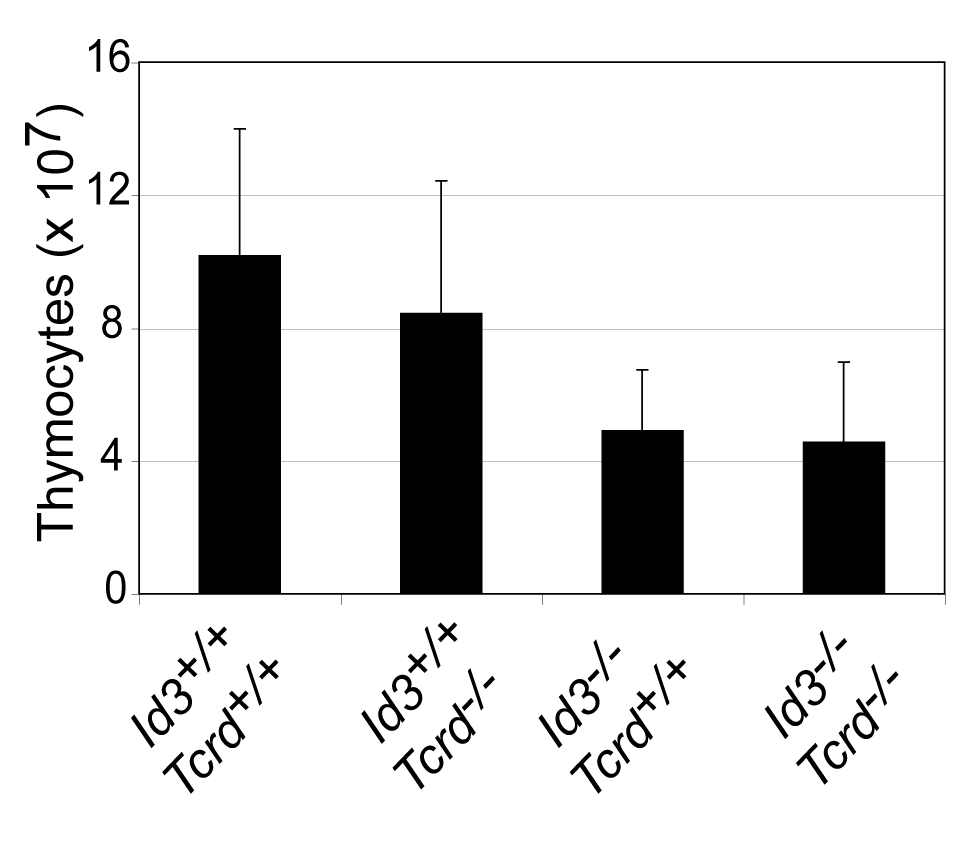

Supplement: Figure S8 — Deletion of Tcrd does not restore thymus cellularity in Id3−/− mice. (A) Total thymocytes numbers in mice of the indicated genotypes. At least 4 mice were analyzed for each genotype. p<0.01 for Id3+/+Sh2d1a−/− or Id3−/−Tcrd−/− compared to Id3+/+Tcrd−/−. (3.93 MB TIF) [file pone.0009303.s008.tif]
